# Why does youth unemployment lead to scarring of depressive symptoms in adulthood? The importance of early adulthood drinking

**DOI:** 10.1177/14034948231208472

**Published:** 2023-12-28

**Authors:** Anne HammarströM, Christopher Bean, Ronnie Pingel, Urban Janlert, Hugo Westerlund, Per Olof Östergren, Pekka Virtanen

**Affiliations:** 1 IMM, Karolinska Institutet, Sweden; 2 Department of Epidemiology and Global Health, Umeå University, Sweden; 3 School of Psychology, University of Adelaide, Australia; 4 Department of Statistics, Uppsala University, Sweden; 5 Stress Research Institute, Stockholm University, Sweden; 6 Social Medicine and Global Health, Department of Clinical Sciences in Malmö, Lund University, Sweden; 7 Faculty of Social Sciences, Tampere University, Finland

**Keywords:** Youth unemployment, scarring, alcohol drinking, life-course epidemiology, depressive symptoms

## Abstract

**Aim:**

The aim of the paper is to analyse if alcohol consumption could explain the scarring effect of youth unemployment on later depressive symptoms.

**Methods:**

The analyses are based on the 24-year follow-up of school leavers in a municipality in Northern Sweden (the Northern Swedish Cohort). Four-way decomposition analyses were performed to analyse if alcohol use at age 30 years could mediate and/or moderate the effect of youth unemployment (ages 18/21 years) on depressive symptoms in later adulthood (age 43 years).

**Results:**

Excessive alcohol use at early adulthood (age 30 years) mediates 18% of the scarring effect of youth unemployment on depressive symptoms in later adulthood. The scarring effect was seen among both those with and without excessive alcohol use.

**Conclusions:**

**Youth unemployment leads to poor mental health later in life and part of these relations are explained by excessive alcohol consumption in early adulthood. Policy interventions should target the prevention of youth unemployment for reaching a lower alcohol consumption and better mental health.**

## Introduction

There are not only direct effects of unemployment on labour market conditions and the financial situation of individuals, but also long-term negative effects on their labour market careers [1]. Unemployment experiences have been related to increased risks of downward occupational mobility and wage penalties [2], consequences usually named unemployment scarring.

Unemployment may also cause scars in the medical sense of the word. It is well known that exposure to unemployment can cause direct negative health effects [3, 4]. However, the damage may not heal at the end of the unemployment spell, but the recovery may remain incomplete, or the exposure may leave a susceptibility to worsening mental health at future stressful life events [5, 6]. The severity of the scar may depend on the timing of the unemployment exposure [6, 7]. During the life course there may be periods of sensitivity for obtaining scars that remain independently of possible later exposures to unemployment [8]. Emerging adulthood may be such a period [9] when frictions in school-to-work transition, such as youth unemployment, are of importance [10]. The concept of scarring in public health builds on a combined ecological [11] and life-course approach [8], which postulates that experiences over the individual’s life course are built into the organism and embodied during life [12] as poor health or susceptibility to future health risks.

We have in earlier research demonstrated that unemployment among young people has not only direct negative effects on health, but also negative long-term effects independent of later unemployment. One study [13] identified young age as a sensitive period for scarring of mental health. Supporting the scarring hypothesis, this study showed that high exposure to unemployment during the age window from 16 to 21 years was associated with somatic symptoms in middle age, even when adjusted for later exposure. Also, other studies on mental health [7, 14] and blood pressure [15] support the concept of scarring as the embodiment (both biologically and psychologically) of early unemployment experience into the risk of immediate poor health but also into susceptibility to future health risks.

It is of major importance to increase the understanding of why youth unemployment can cause health scarring. From a theoretical point of departure, scarring may be understood in the frame of the cognitive activation theory of stress [16]. Unemployment may lead to poor coping, with hopelessness and deteriorated health behaviours as a result, which can remain embodied as future sensitivity to or as future coping with stress. Thus, deteriorated health behaviours such as excessive alcohol consumption may be an explanation. So far, no studies have been found in the field.

The aim of this study is to analyse if alcohol consumption could explain the scarring effect of youth unemployment on later depressive symptoms.

## Methods

### Sample

The Northern Swedish Cohort (NoSCo) consists of all pupils (*n*=1083, 53% male pupils) completing their final year of compulsory schooling in 1981 in a middle-sized town in Northern Sweden. The participants gave informed verbal consent to participate. Aged 16 years at baseline, participants were followed up at ages 18, 21, 30 and 43 years. Questionnaires were completed at each stage; a summary of data collection items and procedures has been published in a cohort profile [17]. Attrition is low; of those alive (*n*=1071) at the latest follow-up in 2008, *n*=1010 (94%) continued participation. Complete data for the current study were available for 962 participants. The participants were requested to indicate their consent by answering the questionnaires. Ethical approval was provided by the Swedish Ethical Review Authority (no. 2020-01950).

### Measures

#### Exposure

Youth unemployment (ages 18–21 years) was determined from a matrix at age 21 years about labour market status during the spring term, summer, and autumn term during the last 3 years. Cumulative unemployment was dichotomised into 3 months or more (‘exposed’) versus less than 3 months (‘not exposed’).

#### Mediator: Alcohol use in early adulthood (age 30 years)

Participants reported frequency of drinking occasions (on a 5-point scale) and their average intake of beer (number of bottles), wine (number of glasses), and strong alcoholic beverages (number of drinks) on each occasion. Alcohol consumption in litres per year of absolute alcohol was computed based on the reported amount and frequencies of intake [18]. ‘High’ alcohol use was defined as the 90th percentile for our sample (5.65 litres/year of pure alcohol). High alcohol use was found among 15.8% of men and 4.1% of women.

#### Outcome: Depressive symptoms (age 43 years)

A previously validated index of six self-reported symptoms during the previous 12 months was used: poor appetite, general tiredness, concentration difficulties, sleep problems, feeling down/sad, and feeling dejected about the future [19]. A scale from 0 to 2 was constructed and used continuously, with higher scores indicating worse depressive symptoms.

#### Covariates: Parental working class belonging

Participants reported the occupation of both parents at age 16 years, and these were classified according to the standard of that time coded as ‘working class’ or ‘non-working class’. Parental working class belonging was categorised on a scale from 0 (neither parent ‘working class’) to 2 (both parents ‘working class’).

Gender was analysed as girls and boys according to the school register at age 16 years.

Depressive symptoms at age 16 years were defined in the same way as at age 43 years.

#### Sensitive period

According to the models of life-course epidemiology [8], we define a sensitive period as a limited time window when exposure has a stronger effect on later health outcomes than it would at other times. To distinguish this model from accumulation, we controlled for later unemployment, measured as the accumulated matrix-reported time in unemployment during ages 31 to 43 years. The variable was dichotomised in the same way as the exposure variable into 3 months or more (defined as ‘exposed’) versus less than 3 months (defined as ‘not exposed’).

### Statistical analyses

To facilitate the simultaneous assessment of mediation and interaction, four-way decomposition [20] was applied, which yields itemised estimates for the four potential contributors:

Controlled direct effect (CDE): The effect of youth unemployment on depressive symptoms at age 43 years, while controlling for alcohol consumption at age 30 years.
Interaction (INTref): The combined effect of youth unemployment and high alcohol use on depressive symptoms attributable to interaction but not mediation.
Mediated interaction (INTmed): The combined effect of youth unemployment and alcohol consumption on depressive symptoms that is attributable to both interaction and mediation, that is, the mediated interaction.
Pure indirect effect (PIE): The effect of youth unemployment on depressive symptoms that is purely mediated by alcohol consumption.

Adjustments for covariates were made in two models, first gender and parental working class belonging and depressive symptom score at age 16 years, second later unemployment exposure was added in the model. Analyses were made using Stata v.15 using the med4way macro.

## Results

Table I presents descriptive statistics for the whole sample, as well as stratified by gender and exposure. Around one in seven (13.7%) of the sample were classified as being exposed to youth unemployment. The main gender differences are seen in alcohol use and depressive symptoms scores: on average, men reported more than twice the amount of alcohol that women drank at age 30 years, while women reported higher depressive symptoms at age 43 years. These differences were largely expected based on previous findings, and informed the decision to control for gender as a confounder in the adjusted models. The group exposed to youth unemployment tended to have higher depressive symptoms – both at baseline (age 16 years) and follow-up (age 43 years), more often reported having working class parents, and higher alcohol use at age 30 years.

**Table I.** Descriptive statistics for study variables (*N*=962).

	Whole sample (*n*=962)		Men (*n*=495)		Women (*n*=467)		Men–women diff.	No youth unempl. ≥3 months (*n*=830)		Youth unempl. ≥3 months (*n*=132)		Youth unempl. status diff.
	*M* (SD)	*n* (%)	*M* (SD)	*n* (%)	*M* (SD)	*n* (%)	*M* (Sig.) ^a^	*M* (SD)	*n* (%)	*M* (SD)	*n* (%)	*M* (Sig.)^a^
**Age 1**												
Youth unemployment												
None or <3 months		*830* (86.28)		*430* (86.87)		*400* (85.65)						
≥3 months		*132* (13.72)		*65* (13.13)		*67* (14.35)	(0.584)					
Age 16 depressive symptoms	0.48 (0.30)		0.41 (0.29)		0.56 (0.28)		–0.14 (<0.001^***^)	0.47 (0.29)		0.54 (0.31)		–0.06 (0.022^*^)
Parental working class belonging^b^												
No working class (0)		*294* (30.56)		*148* (29.90)		*146* (31.26)			*268* (32.29)		*26* (19.70)	
One working class (1)		*323* (33.58)		*162* (32.73)		*161* (34.48)			*290* (34.94)		*33* (25.00)	
Both working class (2)		*345* (35.86)		*185* (37.37)		*160* (34.26)	(0.602)		*272* (32.77)		*73* (55.30)	(<0.001^***^)
**Age 30**												
Alcohol use (litres/year)	2.76 (5.50)		4.06 (7.16)		1.42 (2.14)		(<0.001^***^)	2.59 (5.21)		3.95 (6.98)		–1.36 (0.009^**^)
<90th percentile		*865* (89.92)		*417* (84.24)		*448* (95.93)			*751* (90.48)		*114* (86.36)	
≥90th percentile		*97* (10.08)		*78* (15.76)		*19* (4.07)	(<0.001^***^)		*79* (9.52)		*18* (13.64)	(0.144)
**Age 43**												
Depressive symptoms	0.44 (0.36)		0.36 (0.34)		0.49 (0.38)		–0.11 (<0.001^***^)	0.42 (0.40)		0.56 (0.41)		(<0.001^***^)
Later unemployment (ages 31–43)												
None or <3 months		*855* (88.88)		*439* (88.69)		416 (89.08)			*750* (90.36)		*105* (79.55)	
≥3 months		*107* (11.12)		*56* (11.31)		51 (10.92)	(0.847)		*80* (9.64)		*27* (20.45)	(<0.001^***^)

a *P* values from *t*-test (2-tailed) for continuous and χ^2^ for categorical; ^b^Both parents ‘working class’ coded as 2, one parent ‘working class’ coded as 1, neither parent ‘working class’ coded as 0.

* *P*<0.05; **P<0.01; ***P<0.001.

Table II presents crude and adjusted models to evaluate: the effect of youth unemployment between the ages of 18 and 21 years (i.e. the exposure) on both alcohol use at age 30 years (i.e. the mediator), and depressive symptoms at age 43 years (i.e. the outcome) (Panel 1); the effect of the mediator on the outcome (Panel 2); and the effect of both the exposure and the mediator on the outcome (Panel 3). The ‘Adjusted 1’ models control for gender, baseline depressive symptoms, and parental working class belonging; ‘Adjusted 2’ models additionally control for later unemployment (between ages 31 and 43 years). Exposure to youth unemployment was associated with an average increase of 1.42 litres of pure alcohol per year compared with those not exposed and the association remained after all adjustments (Table II, Panel 1). A similarly robust association is observed between alcohol use at age 30 years and depressive symptoms at age 43 years (Table II, Panel 2). These associations continued to be observed in the mutually adjusted models (Table II, Panel 3). The ‘Final 1’ and ‘Final 2’ models (Table II, Panel 3) detail the specification of the regression models computed to facilitate the four-way decomposition. These models feature the inclusion of the interaction term for the exposure and the mediator.

**Table II.** Models for the mediator and outcome: linear regression for (i) alcohol use at age 30 years, and (ii) depressive symptoms at age 43 years (*N*=962).

	Panel 1			Panel 2			Panel 3				
	Effect of Exposure on (i) Mediator and (ii) Outcome			Effect of Mediator on (ii) Outcome			Effect of Exposure and Mediator on (ii) Outcome				
	Crude	Adjusted 1	Adjusted 2	Crude	Adjusted 1	Adjusted 2	Crude	Adjusted 1	Adjusted 2	Final 1	Final 2
	*b* (95% CI)	*b* (95% CI)	*b* (95% CI)	*b* (95% CI)	*b* (95% CI)	*b* (95% CI)	*b* (95% CI)	*b* (95% CI)	*b* (95% CI)	*b* (95% CI)	*b* (95% CI)
**(i) Model for Alcohol Use, age 30 (Mediator)**											
Youth unempl ^a^	1.36(0.35, 2.36)^***^	1.41 (0.42, 2.40)^**^	1.42 (0.42, 2.42)^**^								
Gender ^b^		–2.69 (–3.49, –1.99)^***^	–2.61 (–3.39, –1.99)^***^								
Age 16 depressive symptoms		0.23 (–0.94, 1.41)	0.24 (–0.94, 1.41)								
Parental working class belonging^c^		–0.00 (–0.42, 0.42)	0.00 (–0.42, 0.42)								
Later unempl. ^a^ (ages 31–43)			–0.15 (–1.24, 0.93)								
**(ii) Model for Dep. Symp., age 43 (Outcome)**											
Youth unempl. ^a^	0.14 (0.71, 0.20)^***^	0.10 (0.03, 0.16)^**^	0.08 (0.02, 0.15)^*^				0.13 (0.06, 0.19)^***^	0.10 (0.03, 0.16)^**^	0.08 (0.02, 0.15)^*^	0.09 (0.02, 0.16)^*^	0.07 (0.00, 0.15)^*^
Gender ^b^		0.09 (0.05, 0.14)^***^	0.09 (0.05, 0.14)^***^		0.09 (0.05, 0.14)^***^	0.09 (0.05, 0.14)^***^		0.09 (0.05, 0.14)^***^	0.09 (0.05, 0.14)^***^	0.09 (0.05, 0.14)^***^	0.09 (0.05, 0.14)^***^
Baseline depressive symptoms (age 16)		0.28 (0.20, 0.35)^***^	0.28 (0.20, 0.35)^***^		0.29 (0.21, 0.36)^***^	0.28 (0.21, 0.36)^***^		0.28 (0.20, 0.35)^***^	0.28 (0.20, 0.35)^***^	0.28 (0.20, 0.35)^***^	0.28 (0.20, 0.35)^***^
Parental working class belonging^c^		0.02 (–0.01, 0.04)	0.01 (–0.12, 0.04)		0.02 (–0.01, 0.05)	0.02 (–0.01, 0.04)		0.01 (–0.01, 0.04)	0.01 (–0.2, 0.04)	0.01 (–0.01, 0.04)	0.01 (–0.02, 0.04)
Alcohol use (age 30)		0.01 (0.01, 0.01)^***^	0.01 (0.01, 0.01)^***^	0.01 (0.00, 0.01)^***^	0.01 (0.01, 0.01)^***^	0.01 (0.01, 0.01)^***^	0.01 (0.00, 0.01)^**^	0.01 (0.01, 0.01)^***^	0.01 (0.01, 0.01)^***^	0.01 (0.00, 0.01)^***^	0.01 (0.00, 0.01)^***^
Later unempl. ^a^ (ages 31–43)			0.15 (0.08, 0.21)^***^			0.15 (0.09, 0.22)^***^			0.14 (0.08, 0.21)^***^		0.15 (0.08, 0.21)^***^
*Interaction*											
Youth unempl. ^a^ × high alcohol use ^d^										0.00 (0.00, 0.01)	0.00 (–0.01, 0.01)

a Dichotomised, ‘yes’ (⩾3 months) coded as 1, ‘no’ (<3 months) coded as 0.

b Women coded as 1, men coded as 0.

c Both parents ‘working class’ coded as 2, one parent ‘working class’ coded as 1, neither parent ‘working class’ coded as 0.

d High alcohol use defined as 90th percentile for sample (5.65 litres of pure alcohol/year).

‘Adjusted 1’ and ‘Final 1’ adjusted for gender, baseline depressive symptoms, parental working class belonging, ‘Adjusted 2’ and ‘Final 2’ additionally adjusted for ‘later unemployment’ (ages 31–43).

* *P*<0.05; **P<0.01; ***P<0.001.

Table III presents the ‘total effect’ of the exposure on the outcome and provides insight into its composition. In the ‘Final 1’ specification, the total effect indicates that those who experienced youth unemployment score on average 0.11 points higher on the depressive symptoms scale at age 43 years, compared with those who did not, given a similar level of baseline depressive symptoms, controlling for gender and parental working class belonging. The CDE was the main contributor with a coefficient (*b*) of 0.10, representing approximately 92% of the total effect. There was no indication of either a reference or mediated interaction between the exposure and the mediator. A PIE of high alcohol use at age 30 years was observed with a coefficient (*b*) of 0.01 or approximately 12% of the total effect. When the respective components are combined, the overall proportion mediated accounts for 15% of the total effect, while the overall proportion attributable to interaction appears negligible.

**Table III.** Four-way decomposition of effect of youth unemployment (ages 18–21 years) and alcohol use (age 30 years) for depressive symptoms at age 43 years (models ‘Final 1’ and ‘Final 2’ as described in Table II) (*N*=962).

	Final 1			Final 2		
	Decomposition			Decomposition		
	*b*	95% CI	Effect proportion	*b*	95% CI	Effect proportion
**Total effect**	0.112**	0.048, 0.176		0.098**	0.034, 0.162	
CDE	0.103**	0.037, 0.170	0.92	0.089**	0.024, 0.155	0.91
INTref	–0.007	–0.036, 0.021	–0.06	–0.009	–0.037, 0.020	–0.09
INTmed	0.003	–0.010, 0.022	0.03	–0.004	–0.010, 0.018	0.04
PIE	0.012*	0.002, 0.025	0.12	0.013*	0.002, 0.025	0.14

CI: confidence interval; CDE: controlled direct effect; INTref: reference interaction; INTmed: mediated interaction; PIE: pure indirect effect.

‘Final 1’ adjusted for gender, baseline depressive symptoms, parental working class belonging^c^; ‘Final 2’ additionally adjusted for ‘later unemployment’ (ages 31–43).

* *P*<0.05; **P<0.01; ***P<0.001.

In the ‘Final 2’ specification, the total effect indicates that those who experienced youth unemployment score on average 0.10 points higher on the depressive symptoms scale at age 43 years, compared with those who did not, given a similar level of baseline depressive symptoms, parental working class belonging, and controlling for gender, as well as later unemployment. The CDE was the main contributor with a coefficient (*b*) of 0.089, representing approximately 91% of the total effect. There was no indication of either a reference or mediated interaction between the exposure and the mediator. A PIE of high alcohol use at age 30 years was observed with a coefficient (*b*) of .013 or approximately 14% of the total effect. When the respective components are combined, the overall proportion mediated accounts for 18% of the total effect, while the overall proportion attributable to interaction appears negligible.

## Discussion

### On the results

Our results show that excessive alcohol use at early adulthood mediates a part of the scarring of youth unemployment on depressive symptoms in adulthood. No interaction effects were found, which means that the scarring mechanisms were seen among both those with and without excessive alcohol use. As shown before [21], youth unemployment per se leads to an increased risk of excessive alcohol consumption, and the findings of this study could indicate that the addictive nature of alcohol makes the increased consumption during youth unemployment remain in early adulthood. Adopting the ‘chain of risks’ model from life-course epidemiology [8], our results indicate that heavy drinking during early adulthood adds to the youth unemployment-related risk of poor mental health later in life. Alcohol could also be used as a kind of self-medication for mental health problems, but there is little or no evidence that such use would have any other long-term effects than alcohol consumption due to other reasons.

Our findings imply that active labour market measures to diminish the rate of youth unemployment could be effective in reducing both excessive alcohol use and depression in adulthood. As to the role of early adulthood drinking for later depressive symptoms, the relatively low figure (14%) of pure indirect effect, however, indicates that intervening specifically against alcohol use among young unemployed individuals would likely have only a limited potential to decrease the risk of later mental health problems.

The complex and reciprocal relations between youth unemployment on the one hand and alcohol consumption as well as depressive symptoms on the other hand have earlier been studied in NoSCo. Youth unemployment was related to increased alcohol consumption, most pronounced among men [18]. The effect of the exposure was stronger than of the selection [18]. A dose–response relation was found between unemployment and depressive symptoms, while the selection effect was quite weak [22].

A multiple response trajectory analysis confirmed high stability in the co-occurrence of depressive symptoms from adolescence until middle age [23]. High alcohol consumption in early adulthood could add to this chain of risk and together with depressiveness be associated with unemployment. This fact does not weaken our earlier and present findings about the scarring effects of youth unemployment.

In developing research on scarring, we have drawn important theoretical and methodological implications. Scarring needs to be conceptualised from the life-course model of sensitive periods [8], which analyses the adult health consequences of exposure to youth unemployment, independent of later exposure to unemployment. If later unemployment is not controlled for, the effect of the exposure during the sensitive period cannot be distinguished from the effect of the accumulation of the exposure or of social chains of risk of exposures during the follow-up. Thus, studies claim that they analyse scarring of unemployment (for example [24] and [25]), but do not control for later unemployment and therefore do not allow separating the effect of a sensitive period from the accumulation of unemployment.

The present study takes a step towards a stricter definition of unemployment scarring by excluding later accumulation of the exposure. In the frames of the life-course model, we control for accumulation of the exposure from the time immediately after measuring the mediator to the time of the outcome. As expected, the results confirm and add to earlier evidence about widespread and long-term health scars due to youth unemployment.

Best possible empirical support for causal inference is needed for policy makers to make use of research findings. The gold standard for empirically supported causality is using randomised controlled trials. However, it would not be possible randomly to assign participants into unemployment. The conditions for interpreting our conclusions as causal (to optimise the theoretical and empirical support for causal assumptions) are theoretically based on the high quality of our empirical data as well as our use of modern analytical causal inference frameworks [26, 27] throughout the analyses including directed acyclic graphs to identify causal pathways and careful selection of confounders (see Supplemental file).

Education, work career and qualifications are interesting topics in relation to our study. Low education in young age is part of a chain of risk, leading to an increased risk of unemployment. Later education and qualifications may decrease the risk of youth unemployment scarring. Future research could analyse whether a successful work career after youth could buffer against the scarring of youth unemployment.

### On the methods

The question of time-varying confounders needs to be addressed. The confounder ‘socioeconomic conditions’ was analysed at age 16 years, while later socioeconomic conditions such as education is not a confounder but rather part of the causal path between exposure and outcome. The same is applicable for the confounder depressive symptoms at age 16 years. Later symptoms are part of the causal chain, rather than time-dependent confounders. The time of measuring baseline depressive symptoms can be discussed. Depressive symptoms should be controlled for before the exposure and we chose the baseline as the most suitable time as no one was unemployed then due to compulsory schooling. Underlying mental disorders were not a confounder as special educational and vocational programmes were directed towards this group. Mediation (alcohol at age 30 years) cannot be analysed at other ages as it should be measured after the exposure and before the outcome.

The timing of measuring the exposure is also important. In earlier publications, we have identified unemployment in youth as compared with later in life as a sensitive period in relation to mental health scarring [6, 14]. In addition, it would make the analyses too complicated to have both four-way decomposition, confounders at age 16 years and to control for effects of unemployment at other ages.

When NoSCo was started in 1981 there was a lack of validated measures of mental health problems among adolescents. Single item questions were used rather than with present composite measures. We have validated our measure of depressive symptoms and found good internal consistency and factorial invariance across the different follow-ups [19].

It could be discussed whether these symptoms reflect mental problems or if they are part of everyday life. According to the psychiatric literature, there seems to be a congruence between self-rated internalised symptoms and clinical diagnoses of major depressive disorder [28, 29]. Thus, these symptoms are regarded as being part of psychiatric diagnoses.

NoSCo has been shown to be largely representative of Sweden in relation to demographics, alcohol consumption and health status [17], with higher exposure to unemployment during the 1980s.

The lack of statistically significant interaction may be due to a lack of power. The main limitation with NoSCo is the relatively small sample size. This was also one reason that we did not stratify the results for gender. In Stata, if you set the level of four-way decomposition with ‘high alcohol use’ at different levels for men and women, this would be two separate analyses. Thus, the only way to take gender into account would have been to analyse the data for men and women separately, which would have effectively halved the sample size and left our analyses underpowered.

Our earlier findings have shown many more similarities than differences between men and women in relation to the mental health effects of unemployment [30]. There are, however, large gender differences in alcohol consumption [18].

Thus, a limitation of this paper is the lack of possibilities to include a gender-sensitive measure of alcohol use.

The main strength of NoSCo is the longitudinal design, from before entering the labour market until midlife, with an exceptionally high response rate. The high response rate was due to the energy from the principal investigator to reach everyone as well as thanks to the large interest for the study among the participants. In addition, all who became unemployed directly after compulsory school were included into an interview study, with regular interview follow-ups during the project period. Thus, those with the highest risk of youth unemployment were not lost during the follow-up.

## Conclusions

Excessive alcohol use at early adulthood mediates a part of the scarring effect of youth unemployment on depressive symptoms in adulthood. The scarring effect was seen among both those with and without excessive alcohol use. Policy interventions should target the prevention of youth unemployment for reaching a lower alcohol consumption and better mental health.

## Supplemental Material

sj-docx-1-sjp-10.1177_14034948231208472 – Supplemental material for Why does youth unemployment lead to scarring of depressive symptoms in adulthood? The importance of early adulthood drinking Supplemental material, sj-docx-1-sjp-10.1177_14034948231208472 for Why does youth unemployment lead to scarring of depressive symptoms in adulthood? The importance of early adulthood drinking by ANNE HAMMARSTRÖM, CHRISTOPHER BEAN, RONNIE PINGEL, URBAN JANLERT, HUGO WESTERLUND, PER OLOF ÖSTERGREN and PEKKA VIRTANEN in Scandinavian Journal of Public Health
