# Supplementary figures and images for: Why does youth unemployment lead to scarring of depressive symptoms in adulthood? The importance of early adulthood drinking
Source: Scand J Public Health. 2023 Dec 28;52(8):960–7. doi: 10.1177/14034948231208472 (PMC11626840; doi:10.1177/14034948231208472)

Directed acyclic graph representing the theoretical causal model of the research question.


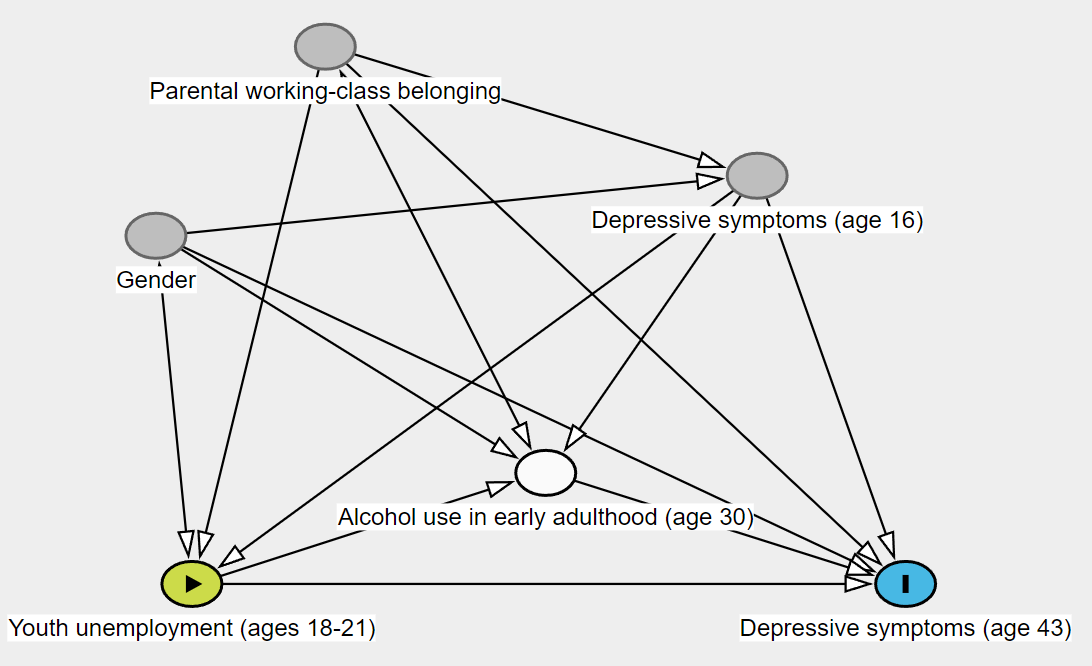

Supplement: sj-docx-1-sjp-10.1177_14034948231208472 – Supplemental material for Why does youth unemployment lead to scarring of depressive symptoms in adulthood? The importance of early adulthood drinking [file sj-docx-1-sjp-10.1177_14034948231208472.docx]
